# Lipidomics Revealed Aberrant Metabolism of Lipids Including FAHFAs in Renal Tissue in the Progression of Lupus Nephritis in a Murine Model

**DOI:** 10.3390/metabo11030142

**Published:** 2021-02-27

**Authors:** Changfeng Hu, Yu Du, Xiaofen Xu, Haichang Li, Qiao Duan, Zhijun Xie, Chengping Wen, Xianlin Han

**Affiliations:** 1 College of Basic Medical Sciences, Zhejiang Chinese Medical University, 548 Bingwen Road, Hangzhou 310053, China; zhudianzhuifeng@163.com (C.H.); xuxiaofen326@163.com (X.X.); lihaichango@163.com (H.L.); duanqiao410@163.com (Q.D.); xzj575@163.com (Z.X.); wengcp@163.com (C.W.); 2 First Clinical Medical College, Zhejiang Chinese Medical University, 548 Bingwen Road, Hangzhou 310053, China; duyuzjtcm@163.com; 3 Barshop Institute for Longevity and Aging Studies, San Antonio, TX 78229, USA; 4 Division of Diabetes, Department of Medicine, Health Science Center at San Antonio, University of Texas, San Antonio, TX 78229, USA

**Keywords:** lupus nephritis, lipidomics, autoimmune reaction, FAHFAs, oxidative stress

## Abstract

Lupus nephritis (LN) is an inflammatory renal disease of patients with systemic lupus erythematosus with lots of immune complexes deposited in kidneys. Accumulated studies have demonstrated the close relationships among dyslipidaemia, inflammation, and autoimmune response, and oxidative stress in the patients. Lipids play numerous important roles in biological process and cellular functions. Herein, shotgun lipidomics was employed to quantitatively analyze cellular lipidomes in the renal tissue of MRL/lpr mice in the progression of LN (including pre-LN and LN state) with/without treated with glucocorticoids (GCs). The levels of cytokines (i.e., TNF-α (Tumor necrosis factor alpha) and IL-6 (Interleukin 6)) in the serum were measured by ELISA (enzyme-linked immunosorbent assay) kits. Renal histopathological changes and C3 deposition in the glomeruli of the mice were also determined. Lipidomics analysis revealed that the ectopic fat deposition and the aberrant metabolism of lipids that were relevant to oxidative stress (e.g., 4-hydroxyalkenal, ceramide, lysophospholipid species, etc.) always existed in the development of LN. Moreover, the anti-inflammatory FAHFA (fatty acid ester of hydroxyl fatty acid) species in the kidney tissue could largely reflect the severity of LN. Thus, they were a potential early biomarker for LN. In addition, the study also revealed that treatment with GCs could prevent the progression of LN, but greatly aggravate the aberrant metabolism of the lipids, particularly when used for a long time.

## 1. Introduction

Systemic lupus erythematosus (SLE), a chronic inflammatory autoimmune disease, is usually prevalent in women of child-bearing age and can affect the majority of the parts of the body, including the heart, skin, liver, blood vessel, and kidneys in particular [1]. Lupus nephritis (LN) is a very common and severe consequence of SLE as it influences ~40–70% of the patients and serves as an important driver of the morbidity and mortality of the disease [2]. So far, the pathogenesis of SLE is mainly attributed to the loss of self-tolerance, aberrant activation of both innate and adaptive immune responses, and autoantibody production. The precise underlying pathological mechanism(s) for LN remain poorly understood, leading to the great challenge of management.

The aberrant metabolism of the lipids contributes to the pathogenesis of LN. Numerous studies have demonstrated that there exists significant alteration of the lipid metabolism in the patients with SLE, including a “lupus pattern” of dyslipoproteinemia [3], aberrant chylomicron metabolism [4], enhanced lipid peroxidation (e.g., higher levels of oxidized low density lipoprotein) [5,6], and other altered profiles of the lipids caused by increased oxidative stress, such as a marked reduction of ethanolamine plasmalogen species, and an obvious increase in the ethanolamine lysoglycerophospholipid (lysoPE) and 4-hydroxyalkenals (4-HNE, the end products of lipid peroxidation) contents [7,8]. Accumulated evidence has supported the “lipid nephrotoxicity hypothesis” that lipid abnormalities could precipitate or/and aggravate glomerular and tubulointerstitial disease through interrupting lipid homeostasis of the renal tissue [9]. These events inevitably result in renal damage, consequently contributing to the progression of LN, since lipids are essential constituents of the cellular membranes, maintaining the cellular functions.

Furthermore, lipids also involve many important biological processes, including signaling transduction, energy storage, and cell growth, differentiation and survival [10]. The recent study conducted by our group also clearly demonstrated that the aberrant lipid metabolism in peripheral blood mononuclear cells from the patients with SLE could lead to dysfunction of the immunocytes, such as the enhanced production of autoantibodies and increased levels of cytokines [11]. The aberrant activation of immune responses and these autoantibody productions could also drastically accelerate the progression of LN. On the other hand, glucocorticoids (GCs) have been widely used for treating nearly every manifestation of SLE for its powerful anti-inflammatory and immunosuppressant effects [12]. It could also produce varieties of adverse effects, especially disturbances of the lipid metabolism. However, it is still unclear whether treatment with GCs could delay/promote the progression of LN. Therefore, the determination of lipid alteration in kidney tissue from the patients with SLE/the lupus murine model could enable us to further understand the pathogenesis and discover novel drug targets.

In the study, to find the aberrant metabolism of lipids contributing to the development of LN, multidimensional mass spectrometry-based shotgun lipidomics (MDMS-SL) technology was performed for class-targeted lipid analysis, including cholesterol, triacylglycerol (TAG), all the classes of phospholipids, various classes of sphingolipids, and branched fatty acid esters of hydroxy fatty acids (FAHFAs) in kidney tissue from a lupus murine model at different ages with/without treatment of GCs. The levels of cytokines TNF-α and IL-6 in the serum samples from the mice were measured by ELISA kits. Histopathological analysis and C3 deposition in glomeruli of the mice from different groups were also performed to assess the severity of LN.

## 2. Results

### 2.1. The Renal Histopathological Changes of MRL/lpr Mice

Accumulated studies have demonstrated that most model mice show renal functional defects at 12 weeks of age [13,14]. The main pathological features include the proliferation of the endothelial and mesangial cells, basement membrane thickening, infiltration of inflammatory cells, and immune complexes (ICs) deposition. Thus, to understand the lipid alteration contributing to the progress of LN, mice were sacrificed at two different time points in the study, at 8 and 14 weeks of age, respectively. Additionally, GCs have been used as the standard treatment of SLE for a long time [15]. Therefore, treatment with GCs was served as the treatment group in the study.

**Figure 1:**
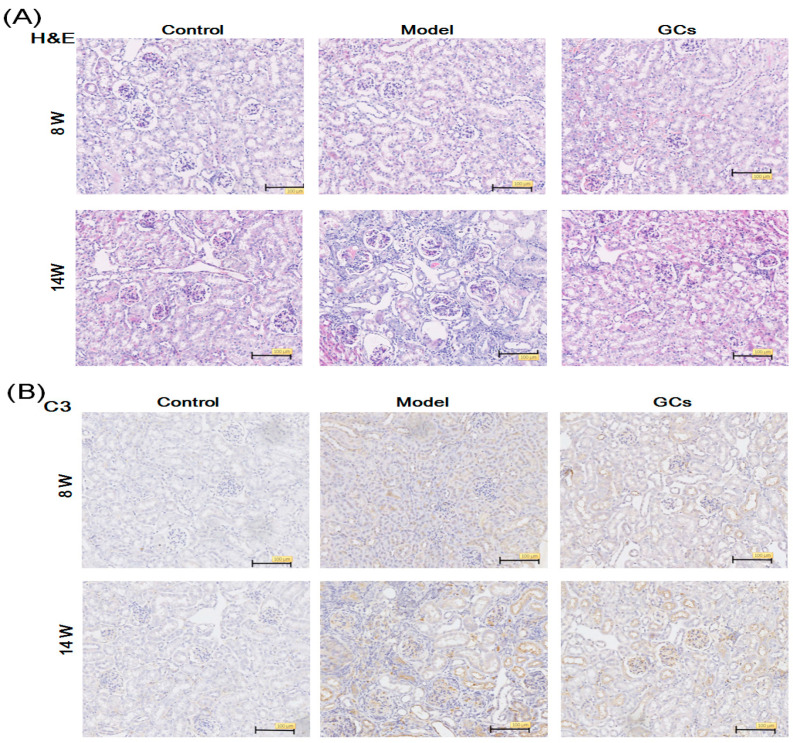
Comparison of renal histopathology of MRL/lpr mice from different groups. Kidney samples of the control (*n* = 4), model (*n* = 4), and glucocorticoids (GCs) (*n* = 4) groups were collected at 8- (pre-lupus nephritis (pre-LN) state) and 14-weeks age (lupus nephritis (LN) state), respectively. Histopathological features of the mouse renal tissues were determined by H&E staining (Panel **A**). The C3 deposition in glomeruli was assessed by immunohistochemical staining (Panel **B**). All representative images were captured under × 100 visual field. H&E denotes hematoxylin and eosin. C3 represents Complement C3.

The analysis of the kidney histopathology showed that at 14 weeks old the model group had obvious glomerular, interstitial, and vascular damage in the kidneys with significant glomerular atrophy, much more mesangial and basement membrane thickening and rupture, mesangial area widening, mesangial matrix increases, and inflammatory cell infiltration, in comparison with those of the controls. The immunohistochemical staining images also showed significant C3 deposition in the glomeruli in the model mice until 14 weeks (Figure 1B). However, these changes and C3 deposition almost did not exist in the kidney tissues from the model at eight weeks of age. Moreover, compared with the model group, these renal damages and the C3 deposition in glomeruli could be greatly alleviated after treatment with GCs (Figure 1A,B).

**Figure 2:**
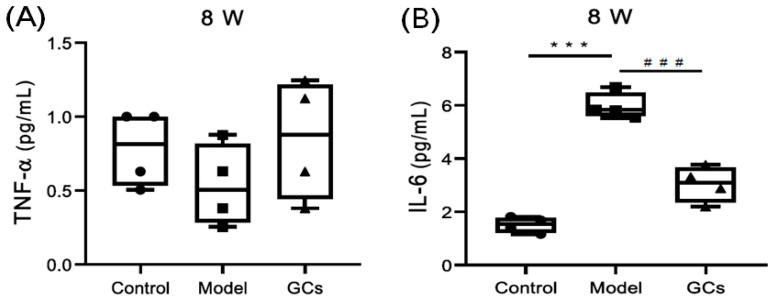
Comparison of concentrations of cytokines in serum samples from MRL/lpr mice of different groups. Serum samples of the control (*n* = 4), model (*n* = 4), and GCs (*n* = 4) groups were collected at eight weeks of age (pre-LN state). The concentrations of cytokines TNF-α (Panal **A**) and IL-6 (Panal **B**) in serum samples of mice from different groups were determined with corresponding ELISA kits, respectively. The filled circle, square and triangle represent the control, model, and GCs group, respectively. The data present means ± SEM (standard error of the mean) from different groups. *** *p* < 0.001 compared with those in the control group, ^###^ *p* < 0.001 compared with those in the model group.

It has been demonstrated that cytokines (e.g., TNF-α and IL-6) implicate in the pathogenesis of LN and their concentrations in the serum could serve as important indexes to reflect the severity of the disease to some extent [16,17]. Therefore, the levels of both these cytokines in sera of mice from different groups were determined. It was found that, compared with that of the control, the concentration of TNF-α in the model did not change significantly (Figure 2A). The levels of TNF-α in the serum samples of the mice from the model group at 14 weeks of age were significantly higher (*p* < 0.001) [11]. Although the level of IL-6 in the model at 8 weeks was substantially elevated (*p* < 0.001), the extent of elevation was much lower that of the model at 14 weeks of age [11]. Incidentally, its concentration of IL-6 could be markedly corrected after treatment with GCs (*p* < 0.001) (Figure 2B).

### 2.2. Cholesterol and TAGs Accumulated in Renal Tissues of MRL/lpr Mice

**Figure 3:**
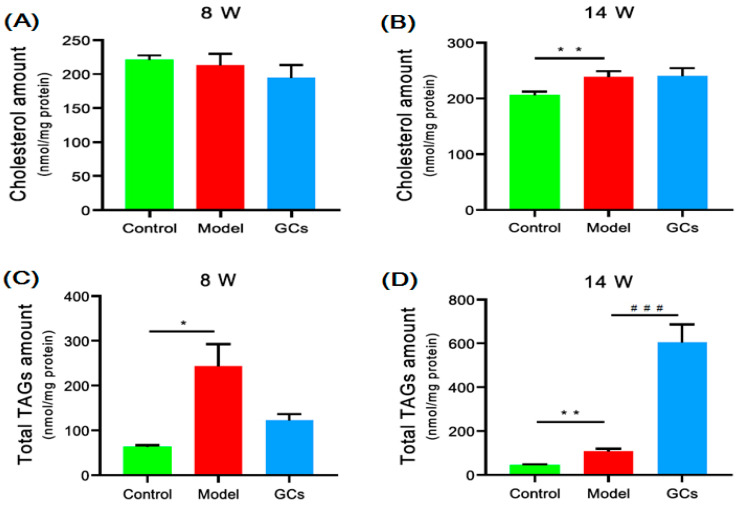
Comparison of the total mass levels of cholesterol and triacylglycerol (TAG) species deposited in renal tissues from MRL/lpr mice at different states of lupus nephritis (LN). The kidney samples of the control (*n* = 4, green), model (*n* = 4, red), and GCs (*n* = 4, blue) groups were collected at 8- (pre-LN state) and 14-weeks of age (LN state), respectively. Lipidomics analysis of cholesterol (Panels **A** and **B**) and TAGs (Panels **C** and **D**) present in lipid extracts of kidneys was conducted by using multidimensional mass-spectrometry-based shotgun lipidomics. The data present means ± SEM from different groups. * *p* < 0.05 and ** *p* < 0.01 compared with those in the control group. ^###^ *p* < 0.001 compared with those in the model group. TAG denotes triacylglycerol.

The lipid nephrotoxicity hypothesis suggested that hyperlipidemia cause a glomerulosclerosis similar to atherosclerosis with numerous lipids, notably cholesterol, accumulated in kidneys and/or other tissues, thereby contributing to the progression of kidney disease [9,18]. Thus, following this line of reasoning, the MDMS-SL technology was performed to determine the level of cholesterol in the renal tissues of the MRL/lpr mice at both 8 and 14 weeks of age. The results of the lipidomics analysis revealed that the total amount of cholesterol in the kidneys from the model group at 14 weeks of age was significantly elevated, leading to an increase from 206.2 ± 3.02 in the controls to 238.0 ± 5.41 nmol/mg protein in the model (an ~15 mol% elevation, *p <* 0.01) (Figure 3B). However, there was no obvious deposition of the cholesterol in the renal tissue of the model at 8 weeks of age. Thus, the results of the cholesterol deposited in the renal tissue during the progress of LN were not consistent with the hypothesis to some extent. In addition, after the treatment of the GCs, the amount of cholesterol in the kidneys was not markedly changed (Figure 3A,B).

To reveal the underlying mechanism(s) leading to LN, the levels of TAG species in kidneys from different groups were also determined using the MDMS-SL technology, since the deposition of TAGs plays a crucial role in the pathogenesis of atherosclerosis. The lipidomics analysis suggested that lots of TAG species accumulated in the renal tissue of the model group even at 8 weeks of age, contributing to increases in the total TAG level from 62.8 ± 3.99 in the controls to 243.3 ± 49.49 nmol/mg protein in the model (*p* < 0.05) (Figure 3C). The total level of the TAG species was increased by ~290 mol%. It should be particularly noteworthy that the degree of TAGs deposited in the kidneys was largely aggravated by the treatment with GCs for 8 weeks (e.g., 108.4 ± 11.03 in the model to 605.1 ± 81.61 nmol/mg protein in the GCs group, *p* < 0.001) (Figure 3D).

### 2.3. Lipidomics Analysis Revealed Significant Increases in Lysophospholipid Species and Elevated Lipid Peroxidation in Kidneys from MRL/lpr Mice

**Figure 4:**
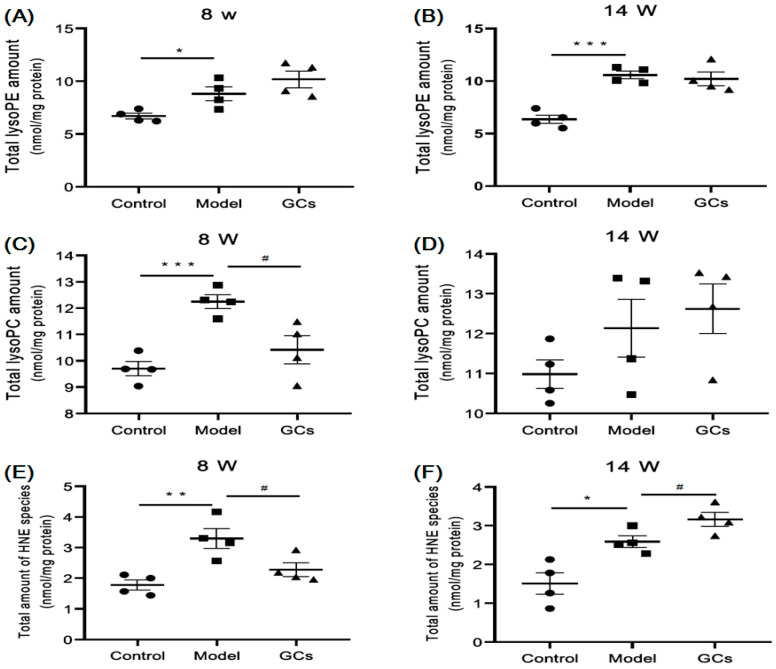
Comparison of the total levels of lysophospholipids and 4-hydroxyalkenal species in renal tissues from MRL/lpr mice at different lupus nephritis (LN) states. The kidney samples of the control (*n* = 4), model (*n* = 4), and GCs (*n* = 4) groups were collected at 8 (pre-LN) and 14 weeks of age (LN), respectively. Lipidomics analysis of lysophospholipids, including ethanolamine lysoglycerophospholipid (lysoPE) (Panels **A** and **B**) and choline lysoglycerophospholipid (lysoPC) (Panels **C** and **D**), and 4-hydroxyalkenal species (Panels **E** and **F**) was performed by the MDMS-SL technology. The filled circle, square and triangle represent the control, model, and GCs group, respectively. The data present means ± SEM from different groups. * *p* < 0.05, ** *p* < 0.01, and *** *p* < 0.001 compared with those in the control group. ^#^ *p* < 0.05 compared with those in the model group. HNE denotes 4-hydroxyalkenal.

To further find the lipid alterations accompanying with the progression of LN, the MDMS-SL technology was performed to quantitatively analyze two of the most important classes of phospholipids (e.g., choline glycerophospholipid (PC) and ethanolamine glycerophospholipid (PE)) in the kidneys from different groups. The results suggested that compared with these of the control, both of the total levels of the PC and PE species did not change in the model group at either 8 or 16 weeks of age (data not shown). In contrast to the minimal change in the total levels of the PC and PE species, the total levels of both lysoPE and choline lysoglycerophospholipid (lysoPC) were markedly elevated in the kidneys in the model even at 8 weeks of age. Specifically, the total levels of the lysoPC and lysoPE species significantly increased from 9.69 ± 0.27 and 6.69 ± 0.27 in the control to 12.25 ± 0.26 and 8.81 ± 0.65 nmol/mg protein in model (*p* < 0.001 and *p* < 0.05), respectively (Figure 4A,C). Incidentally, after treatment with GCs for 8 weeks, the total levels of both lysoPC and lysoPE did not obviously alter in comparison with these of the model group (Figure 4B,D).

Because the elevation of the lysophospholipids is relevant to the increased oxidative stress and enhances the lipid peroxidation present in the serum of SLE patients [19], the levels of HNE species in renal tissues from different groups were also determined using the MDMS-SL technology. As anticipated, the total amount of HNE species was markedly increased in the kidney samples from the model group at 8 weeks of age (i.e., 1.78 ± 0.16 in controls and 3.30 ± 0.33 nmol/mg protein in models, respectively, *p* < 0.01) (Figure 4E). Intriguingly, the degree of lipid peroxidation could be relieved to some extent after treatment with GCs for 2 weeks, whereas it was greatly aggravated after being treated for 8 weeks (Figure 4E,F).

### 2.4. Lipidomics Analysis Revealed Significant Aberrant Metabolism of Sphingolipids in Renal Tissues from MRL/lpr Mice

**Figure 5:**
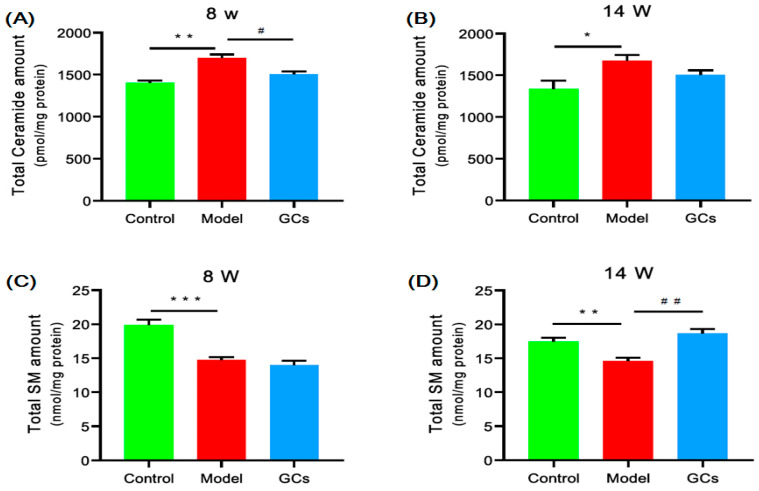
Comparison of the total levels of different sphingolipid species in renal tissues from MRL/lpr mice at pre-lupus nephritis (LN) and LN state. The kidney samples of the control (*n* = 4, green), model (*n* = 4, red), and GCs (*n* = 4, blue) groups were collected at 8 (pre-LN) and 14 weeks of age (LN), respectively. Lipidomics analysis of ceramide (Panels **A** and **B**) and sphingomyelin species (Panels **C** and **D**) present in kidney samples was conducted with the MDMS-SL approach. The data present means ± SEM from different groups. * *p* < 0.05, ** *p* < 0.01, and *** *p* < 0.001 compared with those in the control group. ^#^ *p* < 0.05 and ^##^ *p* < 0.01, compared with those in the model group. SM denotes sphingomyelin.

To reveal the mechanism(s) for the elevated oxidative stress, the levels of ceramide species were also determined with the MDMS-SL approach, since ceramide is an important class of sphingolipids in organisms, implicated in the regulation of oxidative stress [20]. The result of lipidomics analysis showed that the levels of individual ceramide species (especially N16:0 ceramide species) in the model at 8 weeks of age were markedly increased in comparison with those of the controls (*p* < 0.05), contributing to elevating the total level from 1.40 ± 0.03 in controls to 1.70 ± 0.04 nmol/mg protein in models (*p* < 0.01) (Figure 5A). Moreover, after treatment with GCs, the total level of the ceramide species was reduced to some extent (Figure 5A,B).

Due to ceramides serving as the precursors for all complex sphingolipids (e.g., sphingomyelin (SM)), their aberrant metabolism logically results in the corresponding alteration in the SMs. Thus, the MDMS-SL technology was also performed to measure the amount of SMs in the renal tissues from different groups. As anticipated, the total content of SM species was significantly decreased at 8 weeks of age (e.g., 19.90 ± 0.75 in controls and 14.75 ± 0.42 nmol/mg protein in models, respectively, *p* < 0.001). Furthermore, after treatment with the GCs, the aberrant metabolism of the SM species could be corrected, accompanied with the changes in the ceramide species (Figure 5).

### 2.5. Lipidomics Analysis Showed Marked Elevation of FAHFAs Contents in Renal Tissues from MRL/lpr Mice

**Figure 6:**
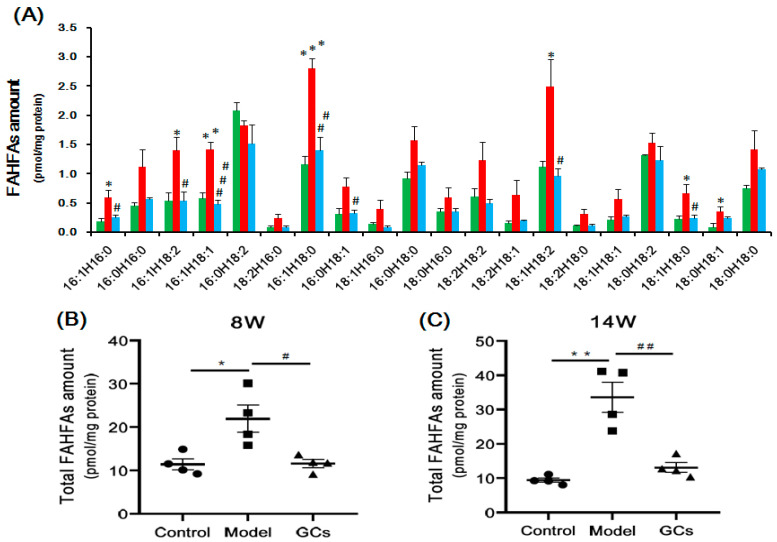
Comparison of the mass levels of fatty acid esters of hydroxy fatty acids in renal tissues from MRL/lpr mice at pre-lupus nephritis (LN) and LN state. The kidney samples of the control (*n* = 4, green), model (*n* = 4, red), and GCs (*n* = 4, blue) groups were collected at 8- (pre-LN) and 14-weeks age (LN), respectively. The levels of individual fatty acid esters of hydroxy fatty acid (FAHFAs) species (Panel **A**) in lipid extracts of kidney samples of mice from different groups at pre-LN state were determined with the MDMS-SL technology. Panels **B** and **C** represent the total amounts of FAHFAs in renal lipid extract from mice of different group at pre-LN and LN state, respectively. The filled circle, square and triangle represent the control, model, and GCs group, respectively. The data present means ± SEM from different groups. * *p* < 0.05, ** *p* < 0.01, and *** *p* < 0.001 compared with those in the control group. ^#^ *p* < 0.05, ^##^ *p* < 0.01, ^###^ *p* < 0.001 compared with those in the model group.

As is well known, chronic inflammation plays a crucial role in the pathogenesis of LN. Thus, the levels of the individual FAHFA species, which possess antidiabetic and anti-inflammatory properties, were also determined using the MDMS-SL technology [21]. The results of the lipidomics analysis showed that the levels of FAHFAs were markedly elevated in the model group in comparison with these of the control at 8 weeks of age, leading to an increase in the total level from 11.43 ± 1.24 in the controls to 21.91 ± 3.14 pmol/mg protein in models, respectively, ~90 mol% increase, *p* < 0.05) (Figure 6A,B). Remarkably, the total concentration of FAHFAs was further elevated with the development of LN (~260 mol% increase, *p* < 0.01) (Figure 6C). Moreover, most noteworthy, the levels of the FAHFA species in the treated groups were markedly reduced compared with these of the model groups at 8 and 14 weeks of age (Figure 6B,C), respectively.

## 3. Discussion

LN is an inflammatory renal disease of the SLE patients with lots of autoantibodies and complement deposition in the kidneys [22]. These ICs would subsequently initiate recruitment and activation of the neutrophils and monocytes to amplify injury [2]. In the study, in addition to the unchanged/minimally changed level of cytokines (e.g., TNF-α and IL-6) in serum samples from the model, the histopathological and immunohistochemical analysis of renal tissues clearly showed that there was less renal damage and immunological deposition of the model group at 8 weeks of age in comparison with those of the model at 14-weeks age (Figure 1 and Figure 2). These results were also consistent with those previously reported [13]. Thus, it was reasonable that the models at 8 and 14 weeks of age were regarded as pre-LN and LN states, respectively.

TAG deposition in the renal tissue might play an important role in the development of LN. The recent research developments that are relevant to the lipid nephrotoxicity hypothesis demonstrated that inflammatory stress accompanying kidney disease modifies lipid homeostasis by increasing cholesterol uptake, inhibiting cholesterol efflux and impairing cholesterol synthesis in peripheral cells, thereby leading to the accumulation of cholesterol in renal, vascular, and possible other tissues, and causing injury [9]. However, the lipidomics analysis showed that cholesterol was not obviously deposited in renal tissue at the pre-LN state. Furthermore, the extent of cholesterol accumulated in the kidneys was relatively low even at the LN state (Figure 3A,B). In contrast to the minimal alteration of cholesterol, TAG species were seriously accumulated in the kidneys in the model at 8 weeks of age. This influences the ectopic fat deposition in the renal tissue in kidneys in the following ways: (1) mechanical stress, for excessive fat around the kidney compresses the renal vein, lymphatic vessels, and the ureters, possibly raising interstitial pressure, impairing sodium exceretion, and consequently inducing albuminuria [23]; and (2) paracrine manner. In particular, the intracellular accumulation of TAGs could induce endoplasmic reticulum stress and subsequent apoptosis [24]. Lipid overload might also overcome the scavenge function of the mesangial cells, in turn, further promoting lipid accumulation, apoptosis, and transformation into foam cells [25]. The excessive accumulation of TAGs could also disrupt mitochondrial function and activates autophagy in the tubular cells, resulting in increased renal gluconeogenesis, tubulointerstitial fibrosis, and lipoapotosis [26]. The combination of these events finally promotes the inflammatory response and cell death, keeping the kidney tissue in a proinflammatory environment with greater macrophage infiltration [27]. Although the exact mechanism(s) for the ectopic fat deposition in the renal tissue is still unknown, it might be correlated with the increased levels of the cytokines TNF-α and IL-6 as previously described [28].

The aberrant lipid metabolism and its metabolites that were relevant with increased oxidative stress could accelerate the development of LN. The elevated concentrations of the HNE and ceramide species clearly suggested that lipid peroxidation caused by increased oxidative stress always existed in the renal tissue during the progression of LN (Figure 4E,F and Figure 5A,B). Numerous studies have revealed the close relationships among oxidative stress, inflammation, and autoimmune responses in patients with SLE [11,29,30]. Specifically, our recent study strongly demonstrated that the aberrant lipid metabolism and its related metabolites could enhance the production of IgG autoantibodies as well as cytokines [11]. In other words, oxidized phospholipids and/or metabolites resulted by increased oxidative stress might serve as important roles of the antigenic epitopes in SLE patients, yielding lots of autoantibodies to oxidized low-density lipoprotein and MDA-modified LDL (malondialdehyde-modified low-density lipoprotein). Moreover, the anticardiolipin antibodies from the SLE patients preferentially recognized oxidized forms of cardiolipin, indicating that varieties of the ICs would be induced and particularly deposited in the renal tissue of the lupus murine model [5]. This process could greatly accelerate the progression of LN. Accompanied with lipid peroxidation, other metabolites (i.e., lysophopholipids) were also yielded [10]. The results of lipidomics analysis in the study also verified this finding (Figure 4A–D). Generally, lysophospholipids with amphipathic characteristics could be toxic to cells/tissues at high concentrations, for they disrupt membrane structure and cause cell lysis. Furthermore, lysophospholipids also display proinflammatory effect, such as promoting the expression of the adhesion molecules, inducing monocyte chemotaxis and proinflammatory cytokine production from macrophages, and enhancing the production of reactive oxygen species [31,32]. Thus, the aberrant metabolism of the lipids caused by oxidative stress could aggravate the development of LN.

FAHFAs, which possess anti-inflammatory property, were a potential early biomarker for LN in kidney tissue. As shown in Figure 6B,C, the total concentration of FAHFA species in the kidney was significantly elevated in the pre-LN state and further elevated with the progression of LN. Moreover, the total levels of FAHFAs were markedly reduced with the severity of LN after treatment with GCs. Therefore, compared with those of other lipids, the total concentration of FAHFAs could correctly reflect the severity of the LN to some extent. The possible mechanism(s) for the anti-inflammatory effect of FAHFAs was blocking the expression of the proinflammatory cytokines (e.g., TNF-α, IL-1β (Interleukin-1β), IL-12 (Interleukin 12), et al.), major histocompatibility complex II, and costimulatory molecules (CD40, CD80, and CD86) required for antigen presentation and T cell activation as previously reported [21].

GCs therapy, particularly for long periods of time, aggravated the aberrant metabolism of the lipids. GCs have been widely used for treating nearly every manifestation of SLE (i.e., LN) and could drastically improve the prognosis of the disease through its powerful anti-inflammatory and immunosuppressant effects [15]. In the present study, the results of the renal histopathology and the alteration of cytokines in the serum sample from the group treated with GCs also strongly supported it to some extent (Figure 1 and Figure 2). However, treatment with the GCs also produced a variety of adverse effects, especially disturbance of the lipid metabolism. After being treated with GCs for 8 weeks, the ectopic fat deposition in the renal tissue was greatly aggravated, as well as increasing the levels of oxidative stress, while these phenomena were not obvious at treatment with GCs for 2 weeks (Figure 3 and Figure 4). These events inevitably resulted in serious consequences. Therefore, particular attention should be paid to the duration of GCs treatment for LN.

In summary, the present study clearly demonstrated that the aberrant lipid metabolism, particularly the ectopic fat deposition and lipid peroxidation, always existed in the development of LN. These results enabled us to further understand the underlying mechanism(s) for the pathogenesis of LN. Moreover, the anti-inflammatory FAHFAs in the kidney tissue, which largely reflect the severity of LN, could be a potential early biomarker for LN. The study also revealed that treatment with GCs could inhibit the progression of LN. However, GCs therapy would greatly aggravate the aberrant metabolism of lipids, particularly when it was used for long time. Thus, more attention should be paid for the duration of GCs treatment for LN.

## 4. Materials and Methods

### 4.1. Materials

GC prednisone (Lot #: P116562) was obtained from Shanghai Aladdin Bio-Chem Technology Co., LTD, China.

All synthetic phospholipids or other lipids, including 1,2-dimyristoleoyl-*sn*-glycero-3-phosphocholine (di14:1 PC), 1,2-dipalmitoleoyl-*sn*-glycero-3-phosphoethanolamine (di16:1 PE), 1-heptadecanoyl-2-hydroxy-*sn*-glycero-3-phosphocholine (17:0 LPC), 1-myristoyl-2-hydroxy-sn-glycero-3-phosphocholine (14:0 LPE), triheptadecenoyl glycerol (T17:1 TAG), (25, 26, 26, 26, 27, 27, 27-d7)-cholesterol, and 4-hydroxy-9,9,9-d3-2(*E*)-nonenal (4-HNE-d3) (100 μg in 200 μL of methyl acetate), used as internal standards were obtained from Avanti Polar Lipid, Inc. (Alabaster, AL, USA), Matreya, Inc. (Pleasant Gap, State College, PA, USA), or Cayman Chemical Co. (Ann Arbor, MI, USA). Additionally, the internal standard FAHFA d4-16:0 ester of 12-OH-18:0 (12-d4 PAHSA) was synthesized as previously reported [33]. All the solvents and chemicals were at least the analytical grade and purchased from Merck KGaA (Darmstadt, Germany), Sigma-Aldrich Chemical Company (St. Louis, MO, USA), or Fisher Scientific (Pittsburgh, PA, USA).

### 4.2. Animal Experiments

The MRL/lpr mouse is a representative murine model of LN, characterizing with massive lymphadenopathy, splenomegaly, numerous autoantibodies, and ICs glomerulonephritis [13]. Female MRL/lpr mice at 6 weeks of age were used in the study. The mice were obtained from the Center Animal House of Zhejiang Chinese Medical University. All the procedures were conducted according to the Ethics Committee for the Use of Experimental Animals at Zhejiang Chinese Medical University. Sixteen of MRL/lpr mice were randomly divided into two groups (8 mice per group): a SLE-water group (the model group) and a SLE-GCs group (the GCs group). Another 8 female MRL/MPJ mice were used as the control group. The GCs group was orally treated with prednisone (5 mg/Kg/day). The dosage of prednisone was adopted depending on the previous experiment [34]. The control and the model groups were fed with the same volume of distilled water. All the mice were maintained in cages at an ambient temperature of 22–25 °C and relative humidity of 60–70% with free access to water and food.

Four mice from each group and the residual were euthanized after treatment for 2 and 8 weeks, respectively. Blood was collected by retro-orbital puncture, further centrifuged (3000 rpm, 15 min, 4 °C) to obtain serum samples. Kidney tissue samples were also removed for histopathological evaluation and lipidomics analysis. The tissue was lavaged with PBS until no blood was present within it, and stored at −80 °C.

### 4.3. Histopathology and Immunohistochemistry

Histopathological evaluation was performed as previously described [14,34]. Mouse kidney tissues were fixed in 4% paraformaldehyde and processed in routine paraffin embedding. Serial sections were cut and stained with hematoxylin and eosin (H&E) and a modified Masson’s trichrome kit. In addition, tissue slides were also stained with anti-C3 antibody after deparaffinization, antigen retrieval, blocking by 3% H_2_O_2_. DAB substrate solution was added to reveal the color of antibody staining after secondary antibody and biotin-streptavidin HRP conjugate incubation. All images were acquired by a Leica microscope equipped with a color camera.

### 4.4. Detection of Cytokines

An aliquot (200 μL) of serum samples of mice from different groups was used for measurement of cytokines (i.e., TNF-α and IL-6) through using detection kits (Jianglai Biotechnology Co., Ltd., Shanghai, China). Determination of cytokines in serum samples was performed according to the manufacturers’ instructions. The concentrations were calculated depending on the standard curves.

### 4.5. Preparation of Lipid Extracts from Kidney Samples

The lipids of individual kidney samples were extracted by using a modified protocol of Bligh and Dyer [35] in the presence of internal standards as previously described [36]. Each lipid extract was resuspended with 2000 µL chloroform/methanol (1:1, *v*/*v*)/mg protein, and stored at −20 °C for lipid analysis. Derivatization of the primary amine in phosphoethanolamine-containing species (such as PE and lysoPE) with fluorenylmethoxycarbonyl chloride and FAHFAs with *N*-[4-(aminomethyl)phenyl]pyridinium (AMPP) was conducted according to the previously reported methods [37,38]. Individual lipid species including FA isomers and regioisomers were identified using multidimensional MS analysis [39,40].

### 4.6. Lipid Analysis, and Data Processing and Analysis

Lipidomic analysis of kidney samples was conducted with a triple-quadrupole mass spectrometer (Thermo TSQ Quantiva) equipped with an automated nanospray ion source (TriVersa NanoMate, Advion Bioscience Ltd., Ithaca, NY, USA) as previously reported [41]. To prevent possible lipid aggregation, the solutions of lipid extracts were diluted in CHCl_3_/MeOH/isopropyl alcohol (1:2:4, *v*/*v*/*v*) prior to direct infusion. All mass spectral data were acquired by different customized sequence subroutines operated under Xcalibur software (Xcalibur 3.0, Thermo Fisher Scientific Inc., San Jose, CA, USA). Data processing was performed according to the previous method [39]. All data were presented at mean ± SEM unless otherwise indicated. Kolmogorov–Smirnov test was used to check the normality of each group of variables. All *p*-values were greater than 0.05, indicating that the data follow the normal distribution. Statistical significance between the groups was determined by Student’s unpaired *t*-tests, where * *p* < 0.05, ** *p* < 0.01, and *** *p* < 0.001.

## Data Availability

The data presented in this study are available in the article.

## Abbreviations

FAHFAs	fatty acid esters of hydroxy fatty acids
GCs	glucocorticoids
4-HNE	4-hydroxyalkenals
ICs	immune complexes
SLE	systemic lupus erythematosus
LN	lupus nephritis
lysoPC	choline lysoglycerophospholipid
lysoPE	ethanolamine lysoglycerophospholipid
MDMS-SL	multidimensional mass spectrometry-based shotgun lipidomics
PE	ethanolamine glycerophospholipid
PC	choline glycerophospholipid
SM	sphingomyelin
TAG	triacylglycerol
